# KIR2DL4 (CD158d): An activation receptor for HLA-G

**DOI:** 10.3389/fimmu.2012.00258

**Published:** 2012-08-20

**Authors:** Sumati Rajagopalan, Eric O. Long

**Affiliations:** Laboratory of Immunogenetics, National Institute of Allergy and Infectious Diseases/National Institutes of HealthRockville, MD, USA

**Keywords:** NK, KIR, HLA-G, pregnancy

## Abstract

KIR2DL4 is an unusual killer cell immunoglobulin-like receptor (KIR) family member in terms of its structure, expression, cellular localization, and signaling properties. The most conserved KIR in evolution, it is referred to as a framework KIR gene and is expressed by all natural killer (NK) cells and a subset of T cells. Although it has a long cytoplasmic tail that is typical of inhibitory KIR, engagement of this receptor results in the activation of NK cells, not for cytotoxicity, but for cytokine and chemokine secretion. Unlike all other KIRs, which are expressed on the surface of NK cells, KIR2DL4 resides in endosomes. It signals from this intracellular site for a proinflammatory and proangiogenic response, using a novel endosomal signaling pathway that involves the serine/threonine kinases DNA-PKcs and Akt. The only known ligand of KIR2DL4 is HLA-G. Soluble HLA-G accumulates in KIR2DL4^+^ endosomes. Unlike classical HLA molecules that serve as ligands for other KIR family members, in healthy individuals, HLA-G expression is restricted to the fetal trophoblast cells that invade the maternal decidua during early pregnancy. Since NK cells constitute the predominant lymphocyte subset at this site, the proinflammatory/proangiogenic outcome of the interaction between KIR2DL4 and soluble HLA-G supports a role for KIR2DL4 in the extensive remodeling of the maternal vasculature during the early weeks of pregnancy.

## INTRODUCTION

The killer cell immunoglobulin-like receptor (KIR) family of activating and inhibitory receptors expressed by natural killer (NK) cells regulates their responsiveness to signals received from their environment. NK cells respond to contact with other cells including tumor cells or virus-infected cells, and to soluble mediators such as cytokines, chemokines, or other soluble ligands. KIR regulate the activation state of NK cells upon recognition of their major histocompatibility complex (MHC) ligands on the surface of target cells (Long, 2008). There is genetic evidence that combinations of KIR and MHC genes contribute directly to different aspects of immunity, such as resistance to infections, susceptibility to autoimmune diseases, transplantation outcomes, and disorders of pregnancy (Parham, 2005).

KIR2DL4 (CD158d), the anchor gene in the middle of the KIR complex, is characterized by its low polymorphism, high degree of conservation among primates, and by its central location among the highly variable KIR family members. There are KIR2DL4 alleles with either 9 or 10 consecutive adenines in exon 6, which encodes the transmembrane domain. This 9A/10A transmembrane genetic polymorphism can result in a truncated receptor encoded by 9A, the function of which is unclear (Goodridge et al., 2003). Unlike other KIR genes that display variegated expression among individual NK cells, KIR2DL4 is constitutively expressed by all NK cells and on all KIR haplotypes (Rajagopalan and Long, 1999).

**FIGURE 1:**
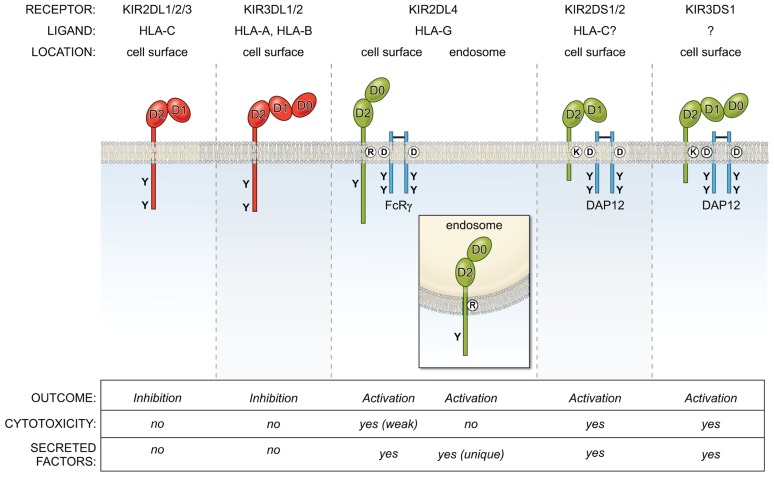
**Overview of the KIR family of inhibitory (red) and activating (green) NK cell receptors.** They bind MHC-I molecules and are located on the cell surface, with the exception of KIR2DL4, which is present in endosomes. The activating receptors interact with the adapters DAP12 or FcRγ via charge-based interactions in the transmembrane region. The KIR2DL/3DL receptors (except KIR2DL4) use ITIM-based inhibitory signaling pathways to block NK effector functions, while KIR2DS/3DS receptors activate NK cells via ITAM-based pathways. In contrast, KIR2DL4 signals from endosomes for a unique secretory response, but does not activate cytotoxicity.

KIR2DL4 is unusual in many other respects, including its structure, localization, and signaling function (**Figure 1**). Unlike KIR with two Ig domains (KIR2DL, KIR2DS) that have a D1 and D2 domain structure, and KIR with three Ig domains (KIR3DL, KIR3DS) with D0, D1, and D2 domains, KIR2DL4 has a hybrid D0–D2 domain structure. Despite having a long cytoplasmic tail characteristic of inhibitory KIR, it carries only a single immunoreceptor tyrosine-based inhibitory motif (ITIM) and displays very weak inhibitory potential (Faure and Long, 2002). Similar to the activating KIR (KIR2DS, KIR3DS) that associate with the immunoreceptor tyrosine-based activating motif (ITAM) containing adaptor DAP12 through a charged amino acid (lysine) in their transmembrane domain, KIR2DL4 has another charged amino acid (arginine) near the top of its transmembrane region. This arginine endows KIR2DL4 with the ability to pair with the γ chain of FcεR1 and to signal for cytotoxicity and cytokine secretion when engaged at the cell surface (Kikuchi-Maki et al., 2005).

## ENDOSOMAL LOCALIZATION

Unlike other KIRs that are present on the NK cell surface, KIR2DL4 is undetectable at the surface of primary resting NK cells isolated from peripheral blood (Goodridge et al., 2003; Kikuchi-Maki et al., 2003; Rajagopalan et al., 2006). Yet, in primary NK cells, engagement of the receptor using soluble agonist antibodies activates robust cytokine secretion but no cytotoxicity (Rajagopalan et al., 2006). This is due to the constitutive localization of the receptor in endosomes, where it resides. However, surface levels can be transiently up-regulated upon culture of these NK cells in interleukin 2 (IL-2; Kikuchi-Maki et al., 2003; Rajagopalan et al., 2006). Weak cytotoxicity upon KIR2DL4 engagement at the cell surface has been detected in IL-2 dependent NK cell lines and IL-2 activated NK cell cultures activated with IL-2 in which a low level of cell surface KIR2DL4 can be detected by flow cytometry (Goodridge et al., 2003; Kikuchi-Maki et al., 2003). In such cells, there is the potential to signal via the adaptor FcεRIγ for cytotoxic responses (Kikuchi-Maki et al., 2005). However, resting NK cells with no apparent surface expression of KIR2DL4 lack the potential to trigger killing, while being fully competent to induce strong cytokine responses (Rajagopalan et al., 2001). Thus, it is important to note that lack of surface KIR2DL4 does not imply lack of receptor expression or the absence of activating function.

In spite of negligible levels of KIR2DL4 on the surface of primary, unstimulated NK cells, activation via soluble antibodies resulted in robust interferon gamma (IFN-γ) secretion in the absence of cytotoxicity (Rajagopalan et al., 2006). This is explained by the finding that at steady state, the majority of KIR2DL4 is intracellular. In cells treated with the K44A dominant negative mutant of dynamin, a GTPase with a central role in clathrin-mediated endocytosis, there was a striking redistribution of the receptor to the plasma membrane, suggesting that KIR2DL4 reaches endosomes by endocytosis (Rajagopalan et al., 2006). KIR2DL4 is localized selectively in early endosomes that are associated with Rab5 and not in other endosomal compartments and perforin-containing cytotoxic granules (Rajagopalan et al., 2006).

This endosomal targeting is unique to KIR2DL4 as other KIR family members are expressed at high levels on the cell surface. Interestingly, it is the Ig domains and not the cytoplasmic tail of KIR2DL4 that control endosomal localization (Rajagopalan, 2010). This was shown by expression of wild-type and chimeric KIR2DL4 receptors in HEK 293T cells and determining their localization by confocal microscopy. A chimera consisting of the extracellular portion of a cell surface NK receptor, gp49B, fused to the transmembrane and tail of KIR2DL4 was not targeted to endosomes and was expressed at the cell surface. In contrast, the Ig domains of KIR2DL4 fused to the transmembrane and tail of gp49B was still targeted to endosomes, showing that the Ig domains were sufficient to target the receptor to endosomes (Rajagopalan et al., 2006).

## SIGNALING FROM ENDOSOMES

Expression of wild-type and chimeric mutants of KIR2DL4 in 293T, and testing for their ability to induce IL-8 secretion proved to be a useful screen for their signaling capability. Such analysis of engineered mutants showed that KIR2DL4 needed to be in endosomes to signal, as chimeric receptors targeted to the cell surface could not signal upon crosslinking at the cell surface. In addition, the cytoplasmic tail of KIR2DL4 was essential for signaling and this endosomal signaling was shown to be independent of both ITIM- and ITAM-mediated signaling (Rajagopalan, 2010). Unlike other KIR at the cell surface that initiate signals through src family kinases such as fyn and lck, signaling by KIR2DL4 was resistant to inhibitors of src family kinases and of phosphoinositide 3 kinase (PI3K; Rajagopalan et al., 2010). Kinase phosphorylation profiling identified the phosphorylation of Akt at serine 473 in response to signaling by KIR2DL4 and this was dependent on its endocytosis (Rajagopalan et al., 2010). The link between regulators of Akt activation and KIR2DL4 was identified by mass spectrometry analysis of associated proteins. This approach revealed DNA-PKcs, a DNA damage signaling kinase as the regulator of Akt. Inhibition of DNA-PKcs blocked Akt phosphorylation, showing that Akt acts downstream of DNA-PKcs (Rajagopalan et al., 2010). In addition, the kinase activity of DNA-PKcs was needed for KIR2DL4 signaling as shown by impaired signaling in the presence of a kinase dead DNA-PKcs. Finally, KIR2DL4 activates a NF-κB response via the canonical pathway to generate a proinflammatory/proangiogenic response. In primary NK cells, the phosphorylation of IκBα and the nuclear translocation of p65, both key events during NF-κB activation, were detected in response to signaling induced by both agonist antibody and soluble HLA-G (Rajagopalan et al., 2010). Phosphorylation of IKKα and IKKβ was detected in an IL-2-activated NK cell line upon antibody crosslinking of surface KIR2DL4 (Miah et al., 2008). In contrast, the DNA-PKcs–Akt–NF-κB signaling pathway occurs in cells that have no detectable surface KIR2DL4. Negative regulation of KIR2DL4 signaling involves Triad3A, an ubiquitin ligase that binds the cytoplasmic tail of KIR2DL4 and promotes its degradation (Miah et al., 2011). Thus, endosomal signaling by KIR2DL4 ensures sustained signals for the secretion of numerous cytokines and chemokines such as IFN-γ, TNF-α, IL-1α, IL-1β, IL-6, and IL-8. An advantage of such intracellular signaling is that it may escape regulation by inhibitory receptors at the NK cell surface.

## EVIDENCE FOR KIR2DL4–HLA-G INTERACTIONS

The only known ligand for KIR2DL4 is the non-classical MHC class I molecule HLA-G. The soluble form of HLA-G may be its natural ligand as it accumulates in KIR2DL4^+^ endosomes and induces endosomal signaling. Soluble HLA-G can be generated either by shedding as a result of the metalloprotease-mediated cleavage of the extracellular domain of the transmembrane isoform HLA-G1 (Park et al., 2004), or by alternative RNA splicing to produce the soluble form known as HLA-G5. Transfected cells expressing a high level of cell surface HLA-G1 were shown to bind recombinant Ig-fusion proteins of KIR2DL4 (Ponte et al., 1999; Rajagopalan and Long, 1999). This binding was blocked by monoclonal antibodies (mAb) to HLA-G and to KIR2DL4 (Rajagopalan et al., 2006).

However, so far, no direct binding of soluble forms of HLA-G to KIR2DL4 has been detected as measured by surface plasmon resonance (Boyson et al., 2002). This could be due to an intrinsic low affinity, as has been the case with activating forms of KIR and their potential MHC-I ligands (Long and Rajagopalan, 2000). Tetrameric HLA-G bound to its ligand ILT4 (CD58d/LILRB2) on monocytic cells, but not to ILT2 (CD85j/LILRB1) or KIR2DL4 on NK cells (Allan et al., 1999). This is likely due to the very low surface expression of ILT2 and KIR2DL4 in primary NK cells. HLA-G can be expressed as monomers or disulphide-linked homodimers on the NK cell surface (Apps et al., 2007). While ILT2 binds preferentially to dimeric HLA-G (Shiroishi et al., 2006), it is not known if KIR2DL4 favors HLA-G monomers over dimers. The crystal structure of dimeric HLA-G and modeling of KIR2DL4–HLA-G interactions indicate that steric constraints would prevent KIR2DL4 from interacting with HLA-G in its dimeric form (Clements et al., 2007). It has been reported that NK cells produced cytokines in the presence of transfectants of 221 cells expressing the HLA-G homodimer, but not with those expressing the HLA-G C42S mutant monomer (Li et al., 2009). Since both monomers and dimers are present on the cell surface of 221 cells expressing wild-type HLA-G, the issue is not resolved. Moreover, Fab fragments of an agonist antibody to KIR2DL4 can activate NK cells indicating that a monomeric ligand can work (Rajagopalan et al., 2006). The interesting question of how KIR2DL4 signaling is initiated with a monomeric ligand remains to be addressed.

Both soluble HLA-G5 and HLA-G shed from the cell surface of transfected cells were accumulated into endosomes that contain KIR2DL4 (Rajagopalan et al., 2006). This was detected by confocal microscopy as co-localization of soluble HLA-G and KIR2DL4 in the same vesicular compartments. There was no accumulation of soluble HLA-C under the same conditions. Evidence for direct binding was obtained by showing that endocytosis of soluble HLA-G was blocked in the presence of a soluble KIR2DL4-Ig fusion protein, while a soluble KIR2DL1-Ig fusion protein did not block uptake of soluble HLA-G (Rajagopalan, 2010). It is likely that transient passage of KIR2DL4 at the cell surface, either by newly synthesized KIR2DL4 or by recycling of endosomal KIR2DL4, is sufficient to capture soluble HLA-G and transport it to endosomes.

Signaling from endosomes offers advantages that are not available to receptors that signal from the cell surface. Endosomal signaling is sustained, lasting minutes to hours in contrast to short-lived cell surface signals. Small endosomal volumes allow for the maintenance of weak receptor–ligand interactions that allow signaling by summation of multiple weak interactions. Endosomes also favor signal maintenance due to slow endosomal sorting and sequestration of signaling complexes in a small volume. Importantly, low *in vivo* levels of ligand can also be overcome by ligand accumulation in endosomes over time. In this way, endosomes provide a platform to optimize weak receptor–ligand interactions due to either affinity considerations or due to limiting amounts of ligand (Scita and Di Fiore, 2010).

## PHYSIOLOGICAL RELEVANCE OF KIR2DL4–HLA-G INTERACTIONS

HLA-G is unusual among HLA molecules in its unique pattern of expression in healthy individuals. Unlike classical MHC-I molecules that are widely expressed on most somatic cells, HLA-G expression is restricted to fetal trophoblast cells at the maternal–fetal interface. However, there is evidence of up-regulation of HLA-G mRNA in response to transformation, neovascularization, inflammation, and infection (Carosella et al., 2008). There is also evidence for increased soluble HLA-G secretion in the serum of patients with malignancies such as melanoma, glioma, ovarian, and breast cancer (Rebmann et al., 2003). Increased levels of circulating sHLA-G have also been detected in viral infections such as HIV-1 (Huang et al., 2010). In the transplantation setting, induction of HLA-G expression correlates with increased transplant acceptance. In healthy individuals, the best-documented evidence of HLA-G protein expression occurs on fetal trophoblast cells that invade the maternal decidua during early pregnancy (Moffett and Loke, 2006). Known receptors for HLA-G are found on NK cells (KIR2DL4 and ILT2) and on monocytic cells (ILT2 and ILT4). All these receptors are present on innate immune cells that comprise the majority of lymphocytes at the maternal–fetal interface in early pregnancy (Moffett-King, 2002).

The presence of soluble HLA-G in human embryo culture supernatants after *in vitro* fertilization (IVF) correlates with successful pregnancy and reduced levels of HLA-G in maternal circulation has been reported in disorders of pregnancy, such as pre-eclampsia and recurrent spontaneous abortion (Rizzo et al., 2010). What is the role of HLA-G and its interactions with NK cells in early pregnancy? The earlier view that HLA-G is tolerogenic by protecting the fetus from attack by maternal NK cells is losing support, as newer findings do not support the role of HLA-G as a tolerance molecule in this context (van der Meer et al., 2004, 2007; Li et al., 2009). In addition to HLA-G, HLA-C, and HLA-E are present on fetal trophoblast cells. Both HLA-C and HLA-E can block NK lysis upon recognition of combinations of inhibitory KIR and CD94/NKG2A receptors expressed by all uterine NK cells (Moffett-King, 2002). Moreover, much of the inhibitory function ascribed to HLA-G is now recognized to be due to HLA-E, since HLA-G expression in cells promotes HLA-E co-expression. This occurs upon binding of the HLA-G signal peptides to the HLA-E peptide-binding groove, resulting in the proper assembly and cell surface expression of HLA-E. In this way, expression of HLA-E on trophoblast cells allows global inhibition of NK cytotoxicity through CD94/NKG2A. Early reports on the effects of HLA-G on NK cells used total peripheral blood mononuclear cells (PBMC) and not purified NK cells to assess NK cell lytic activity (Rouas-Freiss et al., 1997a,b; Riteau et al., 2001a,b). The inhibitory effect of HLA-G was later shown to be on T cells and not on NK cells. Thus, while HLA-G inhibits the proliferation and cytotoxic activity of T cells, it activates decidual NK cells to secrete cytokines and to proliferate (van der Meer et al., 2004, 2007).

**FIGURE 2:**
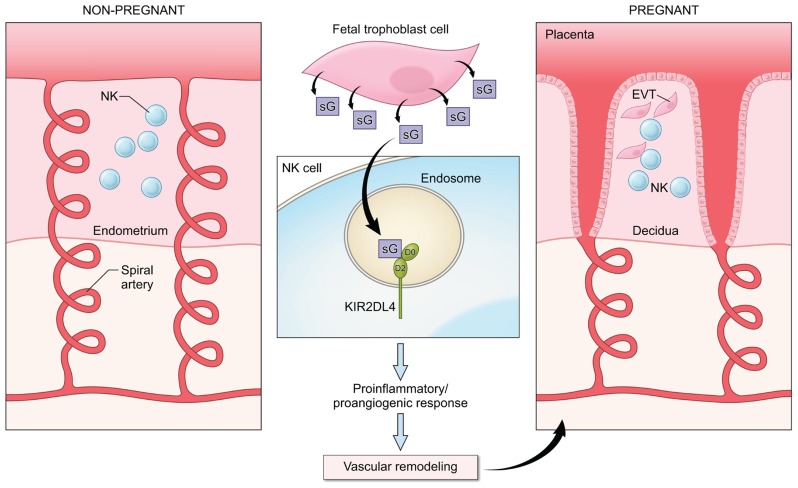
**A potential role for KIR2DL4–HLA-G interactions in early pregnancy.** NK cells are abundant in the non-pregnant endometrium (left panel) in the secretory phase of the menstrual cycle. In early pregnancy (right panel), interactions between fetal extravillous trophoblast cells (EVT) and NK cells in the decidua contribute to the remodeling of the spiral arteries to allow increased blood supply to the fetus. Fetal trophoblast cells secrete soluble HLA-G (sG), which can be endocytosed by KIR2DL4 into endosomes. Endosomal signaling then results in a sustained proinflammatory/proangiogenic secretory response that may promote the vascular changes seen in early pregnancy.

In view of the ability of HLA-G to induce secretion of cytokines and other factors by NK cells and the requirement for soluble mediators to promote the remodeling of the maternal vasculature in early pregnancy, it is likely that HLA-G interacts with KIR2DL4 on NK cells to regulate the production of cytokines and chemokines to promote fetal well being (**Figure 2**). Several genetic association studies point to a useful role for NK activation in early pregnancy and the presence of activating KIR such as KIR2DS1 has been shown to favor reproductive success (Parham and Guethlein, 2010; Chazara et al., 2011). In agreement with this, the production of proinflammatory cytokines in response to HLA-G has been detected in resting peripheral blood NK cells (Rajagopalan et al., 2006), in uterine NK cells isolated during the window of implantation (van der Meer et al., 2004, 2007) and in activated decidual NK cells from first trimester abortions (Li et al., 2009). Analysis of the transcriptional response to activation via KIR2DL4 revealed the up-regulation of a restricted set of chemokines and cytokines, including IFN-γ, TNF-α, IL-1β, IL-6, and IL-8 that can promote vascular remodeling either directly or indirectly by acting on other cell types in the local environment (Rajagopalan et al., 2006; Miah et al., 2008; Li et al., 2009).

## CONCLUSION

KIR2DL4 is an unusual member of the KIR family of receptors. It is expressed by all NK cells and by a subset of T cells. It resides in endosomes and can signal from there through a serine/threonine kinase cascade involving DNA-PKcs, Akt, and NF-κB. This endosomal signaling results in sustained signals for a proinflammatory/proangiogenic response. The ligand of endosomal KIR2DL4 is soluble HLA-G. The selective expression of HLA-G at the maternal–fetal interface, where NK cells are abundant, implies a role for KIR2DL4 during implantation and placentation. Since soluble HLA-G expression is also induced in stress and disease settings, such as tumors, transplantation sites, and during certain infections, it will be important to examine how NK cells and T cells respond to HLA-G at these sites.

## Conflict of Interest Statement

The authors declare that the research was conducted in the absence of any commercial or financial relationships that could be construed as a potential conflict of interest.
